# University of the State of New York

**Published:** 1897-09

**Authors:** 


### AMONG THE COLLEGES.

### UNIVERSITY OF THE STATE OF NEW YORK.

The regents of this college include Hon. Frank S. Black and T. L. Woodruff this year, in place of Levi P. Morton and Charles T. Saxton of last year. Chester Lord is also on the list as succeeding William L. Bostwick of 1896. Frederick A. Putnam has been dropped from the Board of Censors. Dr. Wilfried Lellman has accepted the post of professor of materia medica in addition to the professorship of parasites and parasitic diseases, and Dr. Charles B. Fitzpatrick has been included in the faculty as lecturer on bacteriology. Drs. Goldstein, Cook, and Horpel are no longer among the faculty. For matriculation a student must present a regents' veterinary student certificate, representing at least forty-eight academic counts; a baccalaureate degree from the academic department of a college or university of recognized standing; an academic diploma granted by the regents of the University of the State of New York, or satisfactory proof of having done work considered by the regents as equivalent to the above. Those matriculants who cannot fulfil all the above requirements must pass the regents' examination before the beginning of the second year. Glass's translation of Müller's *Diseases of the Dog* has been added to the list of text-books of this college.

